# Detection of Allergenic Proteins in Foodstuffs: Advantages of the Innovative Multiplex Allergen Microarray-Based Immunoassay Compared to Conventional Methods

**DOI:** 10.3390/foods11060878

**Published:** 2022-03-19

**Authors:** Lisa Tuppo, Ivana Giangrieco, Maurizio Tamburrini, Claudia Alessandri, Adriano Mari, Maria Antonietta Ciardiello

**Affiliations:** 1 Institute of Biosciences and BioResources (IBBR), CNR, 80131 Naples, Italy; lisa.tuppo@ibbr.cnr.it (L.T.); ivana.giangrieco@ibbr.cnr.it (I.G.); maurizio.tamburrini@ibbr.cnr.it (M.T.); 2 Associated Centers for Molecular Allergology (CAAM), 00100 Rome, Italy; claudia.alessandri@caam-allergy.com (C.A.); adriano.mari@caam-allergy.com (A.M.); 3 Allergy Data Laboratories (ADL), 04100 Latina, Italy

**Keywords:** food allergens, IgE binding, IgE-binding inhibition, ISAC test, FABER test, multiplex allergen microarray

## Abstract

Several factors can affect the allergen content and profile of a specific food, including processing procedures often leading to a decrease in allergenicity, although no change, or even an increase, have also been reported. Evaluation of the effectiveness of a processing procedure requires the availability of reliable methodologies to assess the variation in molecules able to induce allergic reactions in the analyzed food. Conventional and innovative strategies and methodologies can be exploited to identify allergenic proteins in foodstuffs. However, depending on the specific purposes, different methods can be used. In this review, we have critically reviewed the advantages of an innovative method, the multiplex allergen microarray-based immunoassay, in the detection of allergens in foodstuffs. In particular, we have analyzed some studies reporting the exploitation of an IgE-binding inhibition assay on multiplex allergen biochips, which has not yet been reviewed in the available literature. Unlike the others, this methodology enables the identification of many allergenic proteins, some of which are still unknown, which are recognized by IgE from allergic patients, with a single test. The examined literature suggests that the inhibition test associated with the multiplex allergen immunoassay is a promising methodology exploitable for the detection of IgE-binding proteins in food samples.

## 1. Introduction

Food allergy is a growing worldwide public health problem affecting 5–10% of the population in developed nations [1]. It has a relevant effect on the well-being of patients and imposes a significant financial burden. Although the estimation of exact costs is not easy and can depend on the examined population, a systematic review of the literature measuring the costs of food allergy reports mean household-level out-of-pocket and opportunity costs of $3339 and $4881, respectively [2]. Actions capable of reducing its impact on human health and the associated social and economic implications are under investigation [3]. Food allergy is a pathological reaction of the immune system triggered by the ingestion of food allergenic proteins in sensitized individuals. The mechanism can be classified, on the basis of the involvement of immunoglobulins E (IgE), in three possible routes: IgE mediated, non-IgE mediated (cell-mediated) or a combination of both [4].

Here we have focused our attention on molecules causing food allergic reactions that are classified as type I hypersensitivities (IgE-mediated), whose symptoms vary from mild localized to severe ones, and include oral allergy syndrome (OAS), urticaria, angioedema, respiratory and gastrointestinal symptoms, anaphylaxis and eczema [5]. They are immediate reactions determined by the production of IgE antibodies towards otherwise innocuous compounds, defined as allergenic proteins. The detection of these proteins allows the estimation of the allergen profile showing the possible allergenicity of the food under investigation [6]. Therefore, the methods useful to detect individual protein molecules can be exploited to achieve this goal and provide the profile of allergenic proteins of a food.

Several factors can affect the allergen content and profile of a specific food, including cultivar, cultivation conditions, climate, post-harvest treatments and processing [7,8]. In particular, food processing often leads to a decrease in allergenicity [9,10,11,12], although no change, or even an increase, have also been reported for foods such as fish [13] and peanut [14,15]. Therefore, the combination of different factors and different types of processing methods can be exploited to obtain foods with lower allergenicity having a lower sensitizing power [16], which can also be consumed by some specific sub-populations of allergic patients. In fact, an increasing number of studies are currently in progress to test the effectiveness of several classic and emerging processing methods in the reduction of the allergenicity of specific foods [17,18]. However, a reliable evaluation of the allergenic proteins in specific foods is of critical importance in order to estimate the efficacy of processing procedures and to select those that are much more effective in the treatment of each food [10]. Several methodological procedures are available and can be used to analyze the allergenicity of a food, and the obtained results strongly depend on the selected methods [19]. 

In this context, a large amount of the literature that has been produced describes several methods useful for allergen detection in foodstuffs. Allergens that can be detected with conventional methods such as ELISA, protein biosensors, DNA-based techniques and mass spectrometry have been reviewed and listed by several authors, although we are citing only a few representatives of them [20,21,22]. In addition, several commercial test kits, which can be exploited by the food industry for the detection of the most common allergens, have been developed and also reported in the literature [20]. At any rate, a systematic and comprehensive review of the available literature on the classical methods useful for allergen detection in foods is not the aim of this paper. In this review, we have rather focused our attention on the use of a new method, the multiplex allergen microarray-based immunoassay, in the detection of food allergenic proteins. In particular, we have analyzed some studies using an inhibition assay on a multiplex allergen biochip, namely the single point highest inhibition achievable assay (SPHIAa) [8,23]. The use of this method to detect and identify allergens in foods has not yet been reviewed in the available literature, probably because it is a quite recent and little exploited procedure. However, this new method could provide a contribution to the allergen control in food processing. An overall description of the classical methods used in this field is here reported with the only aim to make a comparison with the SPHIAa procedure on the multiplex allergen biochip system and to highlight some advantages and disadvantages of the different procedures.

### Food Extract Composition

Allergens cannot be directly analyzed in food samples. It is necessary to prepare food extracts where the allergens are available as soluble molecules that can subsequently be analyzed. In the extracts, the allergenic proteins are mixed with a complex matrix made with many components, which are solubilized during the extraction procedure, including other non-allergenic proteins, sugars, vitamins, salts, polyphenols, etc. The total amount and the relative abundance of each component are strongly dependent on the type of food [24,25] and the features of the starting material [26]. In addition, different extraction protocols can be used and they will provide samples with different features and compositions. For instance, a recent study by Nugraha et al. [27] reported that, compared to low-pH buffers, a higher concentration of proteins was recovered using high-salt or high-pH buffers, revealing more IgE-reactive bands on subsequent immunoblotting. High-salt buffers were also reported to extract with higher efficiency some proteins bound to the cell wall, such as kiwellin and pectin methylesterase [28,29]. The extraction protocols available in the literature can be modified in order to find the best conditions to obtain samples more suited for specific purposes.

In addition, proteins, including the allergenic ones, can interact with the matrix components and this feature can modify their immunological behavior. For instance, the conformation and epitope exposure could change, thus hindering the recovery of allergens or masking the protein epitopes involved in the detection [24,30,31]. Keshavarz et al. [32] demonstrated that parvalbumins purified from mullet and salmon are thermostable proteins. Conversely, the presence of a natural matrix induces thermal instability mainly due to physical (i.e., hydrophobic effect) and chemical interactions (i.e., thiol-disulfide interchange) compromising the extractability and immunodetection. Therefore, even with the same food sample, the matrix components can show variations depending on the protocol used to prepare the extract, thus affecting the results in terms of allergen profile and concentration [33].

Methods used for food processing can include conventional thermal methods and non-thermal ones [13,34,35]. The thermal methods include pasteurization, sterilization, drying and roasting, whereas the others include treatments at high pressure [36], with electric field [37] and irradiation [38], applications of cold plasma [39], enzymatic hydrolysis and fermentation [40]. Protein molecules are sensitive to processing conditions, which can induce modifications such as denaturation [15,41], sometimes associated with aggregation and precipitation, thus generating protein insolubility and low extraction levels [42,43], or can increase their solubility. Protein modifications also include molecule fragmentation [40], cleavage of disulfide bonds [41], formation of covalent intermolecular bonds [42] and Maillard reactions [43]. These modifications can strongly affect the capacity of allergenic molecules to interact with IgE and induce allergic reactions, by reducing or enhancing conformational and sequential epitopes [44]. In fact, following the application of some processing procedures, some allergenic proteins can still be present in the food, although they are no longer able to bind IgE and cause allergic reactions, or their allergenic capacity is changed, due to the generation of new epitopes or to the exposure of otherwise hidden ones. Clearly, the detection of modified allergens, both able and no longer able to bind IgE, depends on several factors, including their extractability associated with solubility and extraction buffers, and on the method used for their analysis. Therefore, depending on the specific aim (detection of IgE-binding or non-IgE-binding allergens) the method to be used can be selected. When the ability of a food processing method to change the allergenicity is under investigation, we can assume that the allergenic proteins, or the DNA coding for allergenic proteins, which are contained in the untreated food, are still present in the processed food, although they could be modified/damaged. To evaluate the effectiveness of the processing procedure we should perform a comparative analysis of allergenicity between treated and untreated food samples. Therefore, we should detect the allergenic proteins able to bind specific IgE before the treatment and analyze whether they can still be recognized after the treatment. In fact, IgE binding represents a precondition for allergenicity, although the sensitization (production of IgE recognizing the allergen) is not a sufficient condition to generate allergic reactions [45,46].

## 2. Classical Analytical Methods Generally Used for Allergen Detection in Foods

Several methods have been used for the detection of allergens in foodstuffs. They include genetic- and mass-spectrometry-based analysis and immunological assays [10,47]. As widely reported [20,21,22], each method has advantages and disadvantages (Table 1), and the choice of one or the another is generally driven by the specific target and/or objective.

### 2.1. DNA Detection

The detection of DNA encoding an allergenic protein or DNA representing a marker of the presence of an allergenic source can be used to reveal potential allergens in foodstuffs. It involves the measurement of DNA amplified by polymerase chain reaction (PCR) following the use of appropriate polynucleotide probes [48,49]. This method is based on molecular biology techniques and it is very specific and sensitive. Although DNA-based methods show these qualities, their accuracy can be strongly affected by the processing of the food product. For instance, several studies have reported that heat treatments and other processing procedures often cause damage, such as DNA fragmentation [50,51], which affects the results of PCR analysis, underestimating its concentration and the health risk declared on the label [52,53].

Actually, the presence of DNA does not mean that the allergenic protein is really available in the food, therefore this is an indirect indicator, which does not reveal the presence of allergenic proteins ready to react with IgE. Furthermore, DNA sequence markers, such as 16S rRNA or mitochondrial gene sequences, can be used to detect the contamination of food products by allergenic sources [54,55] rather than to estimate the amount and the type of allergens in a food sample. In addition, we need to remember that some food components, such as egg white, do not have DNA. Therefore, in this case the detection of allergenic sources by DNA-based methods is not possible. Definitely, this method is not suited to assess the effectiveness of a processing method in the reduction of food allergenicity.

### 2.2. Mass-Spectrometry-Based Technology

Several mass-spectrometry-based methodologies and strategies can be used to identify proteins in food extracts [23,65,66,67,68]. The simultaneous identification and quantification of traces of allergenic proteins in complex mixtures such as processed foods can also be achieved [69,70,71]. Very often the approach known as “bottom-up proteomics” is used. It is based on the generation and analysis of a “peptide mass fingerprinting” obtained by digestion of the proteins with a specific protease, usually trypsin, followed by separation and identification of the fragments by the appropriate mass spectrometer. The mass pattern is then used to search appropriate protein-sequence databases with specific software. The obtained results allow the identification of many proteins/allergens contained in the extract sample with a single experiment. Often mass spectrometry approaches use denatured and fragmented proteins for their identification. Sometimes the extracts are separated by SDS-PAGE before the proteolytic digestion [23]. This procedure often allows the use of not-so-mild extraction conditions, which can include extreme pH and the presence of denaturing and reducing agents. This aspect can represent an advantage because these harsh conditions allow more efficient extraction of, for example, denatured and otherwise insoluble proteins [72]. Therefore, the extracts for mass spectrometry analysis can contain more protein components (including otherwise insoluble or not extracted molecules), compared to those prepared for other types of investigations, such as immunochemical methods. A limitation of mass-spectrometry-based methods can be due to the availability of protein sequences in the searched database. In fact, only the proteins having the corresponding sequence available in the database can be identified in the analyzed sample by mass spectrometry. In addition, mass-spectrometry-based methods allow the detection of proteins even if they were damaged by the processing and are no longer able to bind IgE. Since these methods are not able to discriminate between allergens recognized by IgE and those no longer recognized, they do not appear to be the best choice for a comparative analysis of the effectiveness of a processing procedure in the reduction of food allergenicity. Nevertheless, mass-spectrometry-based methods can contribute to the elucidation of the allergen profile of a food, providing indications about allergens that are not identified by other methods.

### 2.3. Biosensor Technology

A biosensor is a device that measures biological or chemical reactions by generating signals proportional to the concentration of an analyte present in the reaction [56]. It is generally described as a device with three components; namely (i) a biological receptor (enzyme/antibody/cell/nucleic acid/aptamer) that reacts with (ii) a specific analyte, and (iii) a transducer converting the bio-recognition event into a measurable signal [47,57]. On the basis of the type of the transducer used, biosensors can be classified as optical, electrochemical and piezoelectric ones. When the biological receptor is a specific antibody and the analyte is an allergen recognized by that antibody, then the biosensor can be used to detect an allergen in a sample [58]. For instance, biosensors have been used to detect several individual allergens in food samples with high specificity and sensitivity, such as porcine albumin [59], peanut Ara h 1 [60] and Ara h 6 in commercially processed foods [61], Sin a 1 in mustard seeds [62], β-lactoglobulin in dairy products [63] and hazelnut Cor a 14 [64]. Biosensors are sensitive, specific, easy to use, fast and can be used multiple times. In the context of processed foods, depending on the specific molecule considered, biosensors can sometimes contribute to the analysis of an allergen. A disadvantage of this method is that generally only an individual allergen for each test can be analyzed. 

### 2.4. ELISA Assay

Immunochemical methods are based on allergen recognition by a specific antibody, which is generally IgG or IgE. The enzyme-linked immunosorbent assay (ELISA) [73] is the most used immunoassay for allergen detection in foods. It can be performed to evaluate either the presence of antigens or the presence of specific antibodies in a sample. Therefore, it finds application in the detection of antibodies in sera and allergens in food samples. ELISA shows high sensitivity, specificity and good potential for standardization [74,75]. This method requires allergen-specific antibodies, which can be monoclonal or polyclonal ones. ELISA can be performed using two different approaches, namely the “direct ELISA” and the “sandwich ELISA” [49].

The first one is the direct coating approach and it is implemented when the allergen is directly attached to a solid support (usually a polystyrene microtiter plate) by passive adsorption. For instance, a food extract containing many molecules can be attached to the wells and then the presence of a specific allergen can be analyzed by adding a labeled detection antibody (primary antibody) specifically recognizing the searched allergen.

The direct ELISA can also be used to investigate the presence of IgE antibodies in the sera of allergic subjects [76]. In this case, a specific IgE represents the primary antibody recognizing the immobilized allergen and then a secondary labeled antibody is used for detection purposes. This procedure can be also exploited to perform a competitive ELISA (which is an IgE-binding inhibition test) allowing the detection of a specific allergen in liquid samples, such as food extracts [77]. In this case, a specific allergen is incubated with the serum containing IgE antibodies, thus allowing the formation of a complex between the allergen and its specific IgE. The result is that the IgE is no longer available to interact with the allergen immobilized on the solid support of the ELISA plate. Therefore, the IgE binding to the spotted allergen is inhibited and a positive signal (present in the control) will no longer be detected. This missed or reduced signal demonstrates the presence of the allergen, which worked as an IgE-binding inhibitor, in the analyzed sample [78,79].

The sandwich ELISA [80] is carried out by adsorbing the antigen-specific antibody into the wells. This antibody is generally a specific IgG and will capture the antigen contained in the applied sample [81,82], which can be represented by a food extract. Then, a primary antibody (generally IgG) specific for the searched allergen is added, and it binds to the antigen. Next, the complex is revealed by adding a secondary labeled detection antibody. This procedure is more sensitive than the direct one, and it is commonly used when the antigen to be detected is present in small amounts, or its physicochemical properties do not allow sufficient adherence to the wells.

The ELISA assay has been used for many years to detect a great number of different allergens using either in-house-performed tests or an increasing number of commercially available kits. For instance, ELISA commercial kits have been developed for the detection of individual allergens in foods, such as peanut Ara h 1, milk caseins, crustacean tropomyosin, etc. [83,84,85]. Commercial multiplex kits, providing the same high specificity associated with sandwich ELISA and allowing the detection of more than one allergen, are also available and exploit IgG antibodies specific for some foods, such as nuts, egg, milk and gluten [80]. At any rate, comprehensive lists of detectable food allergens and available commercial kits based on the ELISA method have been reviewed and are available in the literature [20,21,22].

ELISA is a fast and sensitive procedure. It is capable of detecting the allergens contained in a sample by revealing the allergenic proteins bearing antigenic epitopes recognized by specific antibodies. Therefore, this immunochemical method cannot detect allergenic proteins that are damaged or unfolded, for instance after the application of processing procedures. A disadvantage of this method is that generally only an individual allergen for each test can be analyzed, although in some cases the detection of more than one allergen can be possible [86].

### 2.5. LFIA

The lateral flow immunoassay (LFIA), also known as a lateral flow immunochromatographic assay, is a simple device that is useful for detecting the presence of a molecule in a food sample [87]. It uses a strip where, in addition to a control, antibodies IgG specific to the target molecule are spotted to form a line. When the liquid sample, which can be a food extract, is loaded in the appropriate space, it will passively flow along the strip. If the searched molecule/allergen is present in the sample, it will reach the specific antibody and will bind it, following the principles of affinity chromatography. Then, a reporter compound will show a signal indicating the formation of the antigen-antibody complex due to the presence of the searched allergen in the analyzed sample. The strip can include one lane (singleplex) or more than one lane (multiplex). LFIA shows advantages and disadvantages similar to those associated with the ELISA assay, but it is quicker than ELISA and has successfully been used for allergen detection in food samples [88,89].

## 3. Multiplex Allergen Microarray-Based Immunoassay for the Detection and the Identification of IgE Binding Proteins

In recent years we have seen the spread of multiplex systems using microarrayed allergens for allergy diagnosis based on the detection of specific IgEs in patients’ sera samples [90,91,92,93] towards already known or new allergens, such as Mor n 3, Ara h 9, Art v 3, Pru ar 5, Act d 5, Act d 11, Pru p 7, Pun g 7, Sola l 7k-LTP and Pun g 14 [5]. The multiplex systems allow the simultaneous measurement of IgE antibodies specific for different individual allergens with the same serum sample, thus improving the diagnostic approach to allergic patients, whose sensitization to, and co-recognition of, other food and inhalant allergens would otherwise remain unknown [94] or might not be well evaluated [95,96].

**Figure 1:**
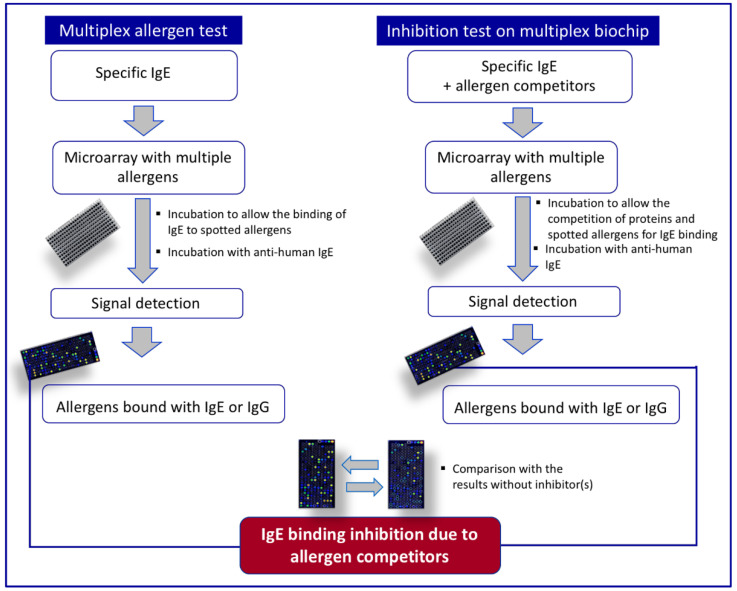
Schematic representation of a multiplex immunoassay, generally used for allergy diagnosis, in comparison with the inhibition assay on a multiplex biochip, such as the single point highest inhibition achievable assay (SPHIAa).

This methodology can also be considered an additional tool useful to detect allergens in a mixture, such as a total protein extract coming from an untreated or processed food sample (Figure 1). In fact, it is possible to perform competition experiments, namely inhibition tests, by preincubating the sera of allergic subjects (or specific antibodies including IgG) with the protein extract. IgE contained in the sera will bind the allergenic proteins of the extract, thus becoming unavailable for the interaction with the microarrayed molecules for which the reactivity with the sera IgE had already been established. Therefore, IgE binding to the proteins in solution is evaluated by recording the residual IgE binding to the allergen(s) spotted on the solid phase [8,97]. Unlike other immunochemical methods, such as IgE immunoblot and ELISA, an advantage of this approach is that the test provides information on many allergens with a single test and gives indications on the identity of the detected IgE-binding proteins.

About 10 years ago, a comparative analysis of the potential allergenicity of 12 apple cultivars, performed with a multiplex biochip-based immunoassay, was reported by Pasquariello and collaborators [8]. This study was focused on the characterization of 10 ancient and 2 commercial widespread apple cultivars and allowed the selection of some hypoallergenic fruits. Their allergenicity was estimated by exploiting the multiplex inhibition method single point highest inhibition achievable assay (SPHIAa) [98] on the ISAC system (Phadia Multiplexing Diagnostics (PMD), Vienna, Austria), by performing IgE- and IgG-binding inhibitions in a single run with a very low amount of allergic patients’ sera and extract preparations. In this study, the version ISAC 103 microarray, containing 103 purified individual allergens spotted on a solid phase, was used (at present the ISAC system includes 112 allergenic proteins, as shown in Table 2, and it is produced by Thermo Fisher Scientific Phadia AB, Uppsala, Sweden). Among the 103 allergens, only one (Mal d 1) was from apple. However, the inhibition experiments also provided information on the presence in the apple extracts of other important allergens, including those belonging to the families of LTP, profilin and thaumatin, by analyzing the inhibition values recorded on homologous molecules from other sources. In addition, indications about the presence in the apple extracts of not yet known apple allergens could be recorded on the basis of the inhibition results on homologous proteins from other allergenic sources, such as 11S globulins, 2S albumins, vicilins, etc.

The IgE-binding inhibition tests with the SPHIAa assay on the allergen multiplex ISAC 103 system was also exploited to investigate the influence of the maturation age on the allergenicity of Parmigiano Reggiano cheese. The study was performed on a population of cow’s milk allergic children. The obtained results showed a variation, associated with the age of cheese maturation, in proteins, peptides and other compounds with different molecular weight and able to bind IgE [99].

A main cause of false positive results in the detection of allergens by immunochemical methods based on the use of specific IgE antibodies is due to cross-reactive carbohydrate determinants (CCDs) bound to the protein molecules. CCDs are present in various allergen sources, such as plant, insect and animal foods, which react with IgE antibodies without inducing relevant clinical symptoms. IgE-binding inhibition to CCDs can be performed on multiplex allergen microarray systems and might allow the detection of signals due to the interference of carbohydrates bound to allergenic molecules [100].

More recently, the SPHIAa assay was applied to the multiplex allergen microarray FABER (Allergy Data Laboratories (ADL), Latina, Italy) [5,97] to obtain information on the allergens contained in food extracts. The SPHIAa method, combined with the FABER technology, represents a forefront tool exploiting a comprehensive panel of 244 allergens (Table 2), namely 122 extracts and 122 purified molecules, including all the most important allergy markers, in addition to exclusive allergens not available in other test systems [5,23,68]. For instance, this method, associated with the use of appropriate sera containing the required IgE, was exploited to perform a comparative analysis of the allergen content in tomato exposed, or not exposed, to nickel (Ni) stress [101]. In particular, the allergenic proteins LTP, profilin, Bet v 1-like protein and TLP were analyzed. The experimental results show that Ni treatment can cause an increase in the allergenic LTP (Sola l 3) and a decrease in profilin (Sola l 1), Bet v 1-like protein (Sola l 4) and thaumatin-like protein. The observed decrease in profilin is in line with the literature reporting a reduction in the concentration of this allergen, assayed with a different method, namely the ELISA test, in the leaves of basil treated with Ni [102].

### Combining Multiplex Allergen Immunoassay with Mass-Spectrometry-Based Methods

**Figure 2:**
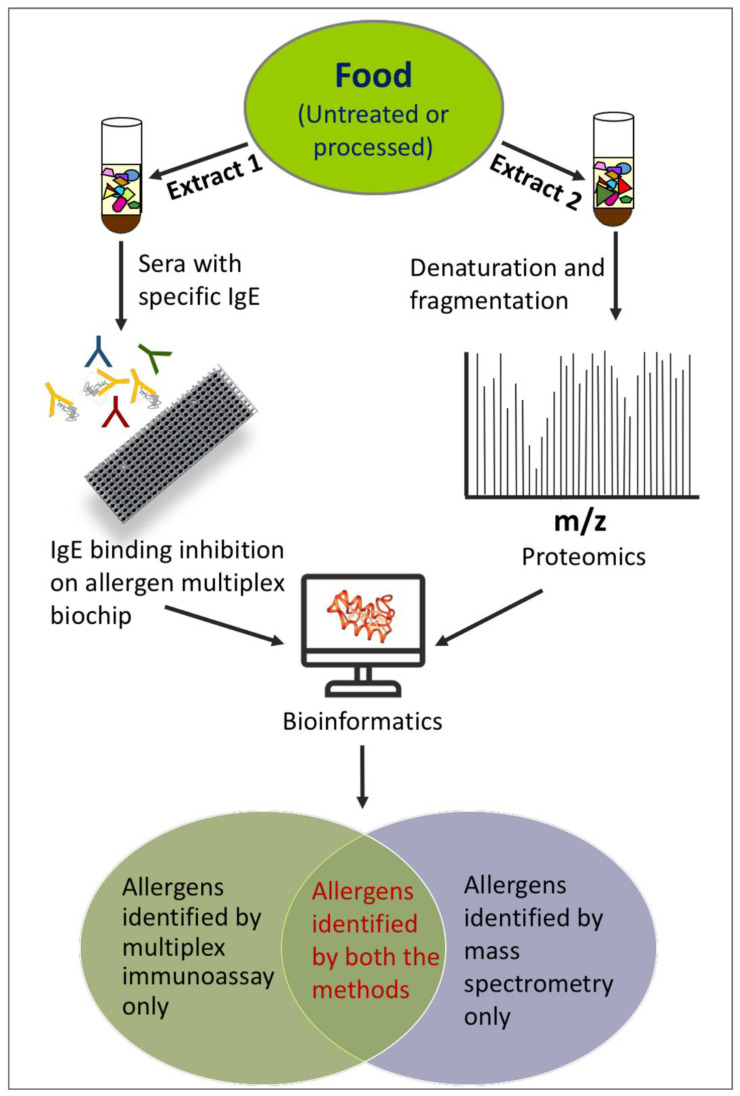
Schematic representation of two procedures used to obtain the allergen profile in a food: inhibition test on a multiplex allergen biochip (on the left) and mass-spectrometry-based procedure (on the right).

A strategy combining the multiplex immunoassay on the FABER biochip and bioinformatics analysis of proteomics data sets (Figure 2) was applied in the study of the potential allergenicity of alfalfa (*Medicago sativa*) leaves [23]. Despite the high economic and agricultural value of this plant, its allergenicity was very poorly known, and no allergens had been identified and registered in the appropriate databases until then. The aqueous extract of the alfalfa leaves was incubated with a pool of sera from allergic patients to allow the competition for IgE binding between possible allergens contained in the extract and those spotted on the FABER biochip. Although no alfalfa samples were available on the FABER biochip, this method allowed the detection of IgE-binding proteins in the analyzed extract by the identification of proteins cross-reacting with plant allergens from several allergen families, such as LTP, thaumatin-like proteins, cysteine proteases, Bet v 1-like proteins, chitinases, and the much more recently identified gibberellin regulated proteins (GRPs) [103,104]. In addition, the absence of structural determinants cross-reacting with seed storage allergenic proteins and with animal allergens was recorded. 

The proteins of alfalfa leaves were also investigated using a mass-spectrometry-based method consisting of in-gel digestion and LC–MS/MS-based proteomics on the protein extract. It allowed the identification of 129 proteins, including the three possible allergens LTP, thaumatin-like and Bet v 1-like proteins. In addition, mass spectrometry allowed the identification of alfalfa proteins homologous to known allergens from other sources, such as hevein, glucanase and chitinases. However, these molecules were not detected by the immunochemical method, namely the SPHIAa assay on the FABER biochip, although homologous allergens were available on the biochip. A possible interpretation of this result is that the extracts used by the two methods contained a different pattern of components. For instance, we cannot exclude that the extraction conditions used to prepare the sample for immunological assays could prevent the solubilization of some proteins that were instead extracted during the preparation of the sample for mass spectrometry. However, proteins belonging to allergen families, such as GRP, were not detected by the mass-spectrometry-based method. Most probably, the protein database searched within the mass spectrometry experiment did not contain GRP sequences, thus preventing its identification. The results obtained in this study highlight that both the methods have limitations, but the combination of the selected methods, namely proteomic experiments, in silico analysis and immunological assay, can produce a much more accurate profile of the allergens contained in the analyzed food.

The same strategy combining immunological tests and bioinformatics analysis of proteomics data sets was applied to the analysis of allergens in three different strawberry-derived vesicle populations [68]. The application of the immunological method, namely the SPHIAa assay on the FABER system, showed that the vesicles carry all the three allergens so far described in strawberry, the Bet v 1-like protein Fra a 1, the LTP Fra a 3 and the profilin Fra a 4. In addition, the immunological method allowed the detection of potential allergens not yet reported in strawberry, such as seed storage proteins, trypsin inhibitor and GRP. However, mass spectrometry experiments allowed the detection of all the three known strawberry allergens in the total strawberry protein extract, whereas only Fra a 1 and Fra a 4 were identified in the vesicle samples. However, mass-spectrometry-based proteomics analysis allowed the detection of several other potential allergens consisting of proteins with sequence similarities to known allergens. They could not be detected by the immunological method because homologous molecules were not available on the allergen biochip used for IgE-binding inhibition experiments.

## 4. Conclusions

The detection of allergens contained in a food, as it is or after processing treatments, is a challenging issue [6], which can be addressed using several methods. Genetic methods allow an indirect detection of allergens. Biochemical methods, such as those based on mass spectrometry, allow a direct detection of proteins belonging to known allergenic families. However, these methods do not provide evidence indicating that these molecules are really recognized and bound by the IgE of allergic patients. Conversely, immunological methods allow the detection of molecules exposing the epitopes recognized by specific IgEs and that are required to induce IgE-mediated allergic reactions. The conventional immunological method that has been highly used for allergen detection in foods is ELISA, which is useful for the detection of a single allergen per test. The multiplex allergen microarray-based system represents an innovative method allowing the detection of many allergens, which are recognized by IgE, with a single test. These advantages do not appear to have been well exploited so far. In fact, the literature reports some examples of the use of multiplex allergen systems to identify IgE binding proteins in food extracts, but it seems that this method has not found application so far for the analysis of allergens in processed foods. Nevertheless, it shows high potentialities for this application. In fact, some features suggest it can be a very useful tool in the analysis of the effectiveness of processing procedures in the reduction of allergenicity.

Clearly, the multiplex allergen microarray-based system is an in vitro test that allows the detection of allergenic proteins and the assessment of their recognition by a specific IgE. However, the assessment of IgE binding is not proof that a protein will really cause an allergic reaction in sensitized patients [105]. The assessment of IgE binding provides useful indications, but when testing the effectiveness of processing methods in the reduction of allergenicity, patient safety requires that the results must always be confirmed by in vivo tests. These tests include prick-by-prick test, skin prick test and the provocation test (double-blind placebo-controlled food challenge, DBPCFC), which remains the “gold standard” for the allergenicity assessment of an untreated or processed food [106].

## Figures and Tables

**Table 1:** Analytical methods for allergen detection in foods.

Methodology	References	Comments	Best Suited for…
DNA-based methods	[48,49,50,51,52,53,54,55]	Identify one or a few allergens for each test It is an indirect method because the presence of DNA is not proof of the allergenic protein presence Some foods do not have DNA	Detection of preselected individual contaminations
Biosensors	[56,57,58,59,60,61,62,63,64]	Generally singleplex detection	Detection of preselected individual allergens for which specific tests, or commercial kits, are available
Mass-spectrometry-based methods	[23,65,66,67,68,69,70,71,72]	Expensive equipment Needs high and specific expertise Best suited for research No discrimination between IgE-binding and non-IgE-binding allergens Does not detect allergens missing in the searched protein database Multiplex detection	Detection of many proteins in the absence of preselection Detection of proteins independently of their ability to be recognized by specific IgE
ELISA	[73,74,75,76,77,78,79,80,81,82,83,84,85,86]	Generally singleplex detection Detection of IgE- and/or IgG-binding allergens	Detection of preselected allergens for which specific antibodies/commercial kits are available
LFIA	[87,88,89]	Generally singleplex detection Does not detect non-IgE- and/or IgG-binding allergens	Detection of preselected allergens for which specific antibodies/commercial kits are available
Multiplex allergen technology	[90,91,92,93,94,95,96,97,98,99,100,101,102]	Multiplex detection with a single test Detection of IgE-binding allergens Detection of still unknown IgE-binding proteins	Detection of many selected and/or unselected allergens with a single test

**Table 2:** List of 122 allergenic extracts and 122 purified allergens contained in the FABER microarray, and 112 purified allergens contained in the ISAC microarray.

Allergen Source	FABER Extracts *	FABER Allergens *	ISAC Allergens *
Gold kiwifruit	Act c (fruit)	Act c 11, Act c chitinase IV	–
Green kiwifruit	Act d (fruit)	Act d 1, Act d 2, Act d 5, Act d 10	Act d 1, Act d 2, Act d 5, Act d 8
Mosquito	Aed c (saliva)	–	–
Onion	All c (bulb)	–	–
Leek	All p (bulb)	–	–
Garlic	All s (bulb)	–	–
Alder	–	–	Aln g 1
Alternaria	–	Alt a 1, Alt a 6.0101	Alt a 1, Alt a 6
Amaranth	Ama cr (seed)	–	–
Ragweed	Amb a (pollen)	Amb a 1	Amb a 1
Pineapple	–	Ana c 2	–
Cashew	Ana o (seed)	Ana o 3	Ana o 2
Duck	Ana p (egg yolk), Ana p (egg white)	_	_
Anisakis parasite	Ani pe (larva)	Ani s 1, Ani s 3	Ani s 1, Ani s 3
Celery	Api g (stalk)	Api g 1.0101	Api g 1
Honey bee	Api m (venom)	Api m 1, Api m 4	Api m 1, Api m 4
Peanut	Ara h (seed)	Ara h 1, Ara h 2, Ara h 3, Ara h 6, Ara h 8.0101, Ara h 9, Ara h agglutinin	Ara h 1, Ara h 2, Ara h 3, Ara h 6, Ara h 8
Horseradish	–	Arm r horseradish peroxidase	–
Mugwort	Art v (pollen)	Art v 1	Art v 1, Art v 3
*Aspergillus*	Asp f (whole body)	Asp r 1	Asp f 1, Asp f 3, Asp f 6
Asparagus	Aspa o (stem)	–	–
Brazil nut	Ber e (seed)	–	Ber e 1
Birch	Bet v (pollen)	Bet v 1.0101, Bet v 2.0101	Bet v 1, Bet v 2, Bet v 4
Common beet	Beta v (leaf)	–	–
German cockroach	Bla g (whole body)	Bla g 1, Bla g 2, Bla g 4, Bla g 5,	Bla g 1, Bla g 2, Bla g 5, Bla g 7
Blomia	Blo t (whole body)	–	Blo t 5
Cow	Bos d (milk), Bos d (muscle)	Bos d 4, Bos d 5, Bos d 6, Bos d 8, Bos d carbonic anhydrase, Bos d gelatin, Bos d lactoferrin	Bos d 4, Bos d 5, Bos d 6, Bos d 8, Bos d lactoferrin
Buffalo	Bub b (milk)	–	–
Camel	Cam d (milk)	–	–
Dog	Can f (epithelium)	Can f 1, Can f 2, Can f 3, Can f 5	Can f 1, Can f 2, Can f 3, Can f 5
Candida	Cand a (whole body)	–	–
Goat	Cap h (milk)	–	–
Chestnut	Cas s (seed)	–	–
Guinea pig	Cav p (epithelium)	–	–
Carob	Cer si (seed)	–	–
Goosefoot	–	–	Che a 1
Quinoa	Que qu (seed)	–	–
Chickpea	Cic a (seed)	–	–
Tangerine	Cit r (fruit)	–	–
Cladosporium	Cla h (whole body)	–	Cla h 8
Hazelnut	Cor a (seed)	Cor a 1.0103, Cor a 14, Cor a 8, Cor a 9	Cor a 1.0101, Cor a 1.0401, Cor a 8, Cor a 9
Common quail	Cot c (egg yolk), Cot c (egg white)	–	–
Hamster	Cri c (epithelium)	–	–
Japanese cedar	Cry j (pollen)	–	Cry j 1
Cantaloupe melon	Cuc m (fruit)	–	–
Cucumber	Cuc s (fruit)	–	–
Cypress	–	Cup a 1	–
Bermuda grass	–	–	Cyn d 1
Carrot	Dau c (root)	–	–
Mites	Der p (whole body)	Der f 1, Der f 2, Der p 1, Der p 2, Der p 10, Der p 23.0101, Der p 7, Der p 9	Der f 1, Der f 2, Der p 1, Der p 2, Der p 10, Lep d 2
European anchovy	Eng e (muscle)	–	–
Donkey	Equ as (milk)	–	–
Horse	Equ c (epithelium), Equ c (milk)	Equ c 3, Equ c myoglobin	Equ c 1, Equ c 3
House dust mite	–	Eur m 2	-
Buckwheat	Fag e (seed)	-	Fag e 2
Cat	Fel d (epithelium)	Fel d 1, Fel d 2	Fel d 1, Fel d 2, Fel d 4
Fennel	Foe v (bulb)	–	–
Strawberry	Fra a (fruit)	–	–
Atlantic cod	Gad m (muscle)	–	Gad c 1
Chicken	Gal d (egg yolk), Gal d (egg white), Gal d (muscle)	Gal d 1, Gal d 2, Gal d 3, Gal d 4, Gal d 5	Gal d 1, Gal d 2, Gal d 3, Gal d 5
Soybean	Gly m (seed)	Gly m 1, Gly m agglutinin, Gly m trypsin inhibitor	Gly m 4, Gly m 5, Gly m 6
Snail	Hel as (muscle)	Hel as 1	–
Rubber tree	Hev b (latex)	Hev b 1, Hev b 10, Hev b 11, Hev b 3.0101, Hev b 5.0101, Hev b 6.02, Hev b 7.02, Hev b 8	Hev b 1, Hev b 3, Hev b 5, Hev b 6.01, Hev b 8
American lobster	Hom a (muscle)	–	–
Human	–	Hom s serum albumin, Hom s lactoferrin	–
Barley	Hor v (seed)	–	–
Walnut	Jug r (seed)	Jug r 2, Jug r 3	Jug r 1, Jug r 2, Jug r 3
Lettuce	Lac s (leaf)	–	–
Lentil	Len c (seed)	–	–
Linseed	Lin us (seed)	–	–
Shrimp	Lit v (whole body)	Lit v 1	Pen m 1, Pen m 2, Pen m 4
Rye grass	Lol p (pollen)	Lol p 1	–
Lupine	Lup a (seed)	–	–
Apple	Mal d (fruit)	Mal d 1.0108	Mal d 1
Common turkey	Mel g (egg yolk), Mel g (egg white), Mel g (muscle)	–	–
Annual Mercury	–	Mer a 1	Mer a 1
European Hake	–	Mer mr 1	–
Mouse	Mus m (epithelium)	Mus m 1, Mus m 4	Mus m 1
Mussel	Myt g (muscle)	–	–
Olive tree	Ole e (pollen)	Ole e 1, Ole e 2	Ole e 1, Ole e 7, Ole e 9
Rabbit	Ory c (epithelium), Ory c (muscle)	Ory c 6	–
Rice	Ory s (seed)	–	–
Sheep	Ovi a (milk), Ovi a (muscle)	Ovi a 6	–
Pellitory	Par j (pollen)	Par j 2	Par j 2
Penicillium	Pen ch (whole body)	–	–
American cockroach	Per a (whole body)	Per a 7	–
Avocado	Pers a (fruit)	–	–
Bean	Pha v (seed)	–	–
Timothy grass	Phl p (pollen)	Phl p 1.0102, Phl p 2.0101, Phl p 5.0101, Phl p 6.0101, Phl p 7.0101	Phl p 1, Phl p 2, Phl p 4, Phl p 5b, Phl p 6, Phl p 11, Phl p 12
Pine nut	Pin p (seed)	–	–
Peas	–	Pis s 3	–
Pistachio	Pis v (seed)	–	–
American sycamore	Pla a (pollen)	Pla a 1	Pla a 1, Pla a2, Pla a 3
Ribwort	–	–	Pla l 1
Mushroom	Ple o (whole body)	–	–
Paper wasp	Pol spp (venom)	–	Pol d 5
Apricot	Pru ar (fruit)	–	–
Almond	Pru du (seed)	–	–
Peach	Pru p (pulp), Pru p (peel)	Pru p 3, Pru p 7	Pru p 1, Pru p 3
Pomegranate	Pun g (fruit)	Pun g 1, Pun g 14, Pun g 5, Pun g 7	–
Oak	Que a (pollen)	–	–
Rat	Rat n (epithelium)	Rat n 1, Rat n 4	–
*Saccharomyces*	Sac c (whole body)	–	–
*Salsola*	–	–	Sal k 1
Salmon	Sal s (muscle)	–	–
Sesame	Ses i (seed)	–	Ses i 1
White mustard	Sin a (seed)	–	–
Common sole	Sol so (muscle)	–	–
Tomato	Sola l (fruit), Sola l (seed)	Sola l 6	–
Eggplant	Sola m (fruit)	–	–
Potato	Sola t (tuber)	Sola t 1	–
Spinach	Spi o (leaf)	–	–
Domestic pig	Sus s (muscle)	Sus s 1	–
Tuna	Thu a (muscle)	–	–
Wheat	Tri a (seed)	Tri a 7k-LTP, Tri a 18, Tri a 28, Tri a gliadin	Tri a 14, Tri a 19.0101, Tri a_trypsin inhibitor
Trichophyton	Tri me (whole body)	–	–
Kamut	Tri tp (seed)	–	–
Squid	Uro du (muscle)	Uro du 1	–
Clam	Ven ga (muscle)	Ven ga 1	–
Wasp	Ves spp (venom)	–	Ves v 5
Grape	Vit v (fruit)	–	–
Corn	Zea m (seed)	Zea m 14	–

* Details about the listed extracts and purified allergenic proteins can be found at the WHO/IUIS website http://allergen.org/ (last accessed on 10 March 2022) and/or at the Allergome website http://www.allergome.org (last accessed on 10 March 2022).

## Data Availability

Not applicable.
